# Review of Diagnostic Modalities for Adrenal Incidentaloma

**DOI:** 10.3390/jcm12113739

**Published:** 2023-05-29

**Authors:** Dominika Okroj, Agata Rzepecka, Przemysław Kłosowski, Anna Babińska, Krzysztof Sworczak

**Affiliations:** Department of Endocrinology and Internal Medicine, Faculty of Medicine, Medical University of Gdańsk, ul. Dębinki 7, 80-211 Gdańsk, Poland; dominika.okroj@gumed.edu.pl (D.O.); agatarzepecka@gumed.edu.pl (A.R.); przemek.k@gumed.edu.pl (P.K.); ksworczak@gumed.edu.pl (K.S.)

**Keywords:** adrenal tumor, incidentaloma, adrenocortical adenoma, adrenocortical cancer, metabolomics

## Abstract

Adrenal incidentalomas are common findings in clinical practice, with a prevalence of up to 4.2% in radiological studies. Due to the large number of focal lesions in the adrenal glands, it can be challenging to make a definitive diagnosis and determine the appropriate management. The purpose of this review is to present current diagnostic modalities used to preoperatively distinguish between adrenocortical adenoma (ACA) and adrenocortical cancer (ACC). Proper management and diagnosis are crucial in avoiding unnecessary adrenalectomies, which occur in over 40% of cases. A literature analysis was conducted to compare ACA and ACC using imaging studies, hormonal evaluation, pathological workup, and liquid biopsy. Before deciding on surgical treatment, the nature of the tumor can be accurately determined using noncontrast CT imaging combined with tumor size and metabolomics. This approach helps to narrow down the group of patients with adrenal tumors who require surgical treatment due to the suspected malignant nature of the lesion.

## 1. Introduction

Adrenal incidentalomas are common findings in clinical practice during imaging studies, with a prevalence of 2.3% in autopsy series and 0.5–4.2% in radiological cases [1]. Nonfunctioning adenomas account for approximately 70% of these cases, but other lesions must also be considered in the differential diagnosis (Table 1) [2,3].

When an adrenal mass is discovered, two important questions must be addressed: whether the tumor is autonomously secreting hormones and whether it is malignant. Patients with functionally active or potentially malignant adrenal masses are typically considered for surgery [4].

During the initial diagnosis, it is recommended to exclude malignant neoplasms [5]. While only 2% of incidentally discovered adrenal masses represent metastasis, the risk is as high as 50–75% when patients have an active or previously diagnosed extra-adrenal malignancy. The adrenal glands are common sites of metastasis from a variety of primary tumors, including lung (56%), breast, kidney, and gastrointestinal tract carcinomas [6].

The aim of this review is to present current diagnostic modalities used to preoperatively distinguish between adrenocortical adenoma (ACA) and adrenocortical cancer (ACC). Proper diagnosis and management are crucial in avoiding unnecessary adrenalectomies, which occur in over 40% of cases [7,8].

## 2. Epidemiology

ACC accounts for approximately 5% of incidentalomas, but its incidence could be up to 12% in surgical studies [2,7,8]. ACC was found incidentally (without any signs or symptoms suggesting hormonal excess or malignancy) in 16% and even up to 43.9% in another study [8,9]. The mean age at diagnosis of ACC is 46–51.2 years, and it affects women more commonly than men [7,8,10,11].

## 3. Imaging Techniques

To distinguish between benign and malignant adrenocortical tumors, three imaging techniques are currently in use. One of the techniques is noncontrast (unenhanced) computed tomography (CT), which is crucial in the initial assessment of adrenal masses [4]. This technique provides information about tissue attenuation, measured in Hounsfield units (HU). On unenhanced CT images, there is an inverse linear relationship between fat concentration and attenuation. Therefore, assessing the lipid content of adrenal masses has become the basis for differentiating between benign (high lipid content) and potentially malignant (low lipid content) adrenal masses [12].

Studies have shown that an attenuation value of 10 HU or less in noncontrast CT is specific for lipid-rich adenomas, indicating that the lesion is unlikely to be malignant. This cutoff value is recommended in the latest European guidelines [4,13,14]. While a high lipid content is a good indicator of an ACA on CT or magnetic resonance imaging (MRI), not all ACAs have a high lipid content. At least 30% of ACAs have low lipid content, resulting in an attenuation value >10 HU. This can cause them to have a similar density to pheochromocytomas and malignant lesions, which reduces the accuracy of this approach [13]. As a result, many patients may undergo additional imaging studies and procedures, which can lead to unnecessary surgeries. In a study by Mantero et al., 69% of resected ACAs were nonfunctioning and did not meet any criteria for surgery [7]. Similarly, Bancos et al. found that 50.3% of surgically removed ACAs were either nonfunctioning or showed only mild autonomous cortisol secretion. They concluded that 43.5% of all participants in the study who underwent adrenalectomy did not require it [8]. The study also discovered that 31.9% of ACAs on noncontrast CT had an attenuation value >10 HU, which resulted in false positives. However, increasing the unenhanced CT attenuation threshold to 20 HU from the recommended 10 HU improved the accuracy of diagnosing ACC while maintaining sensitivity. Specifically, the specificity increased from 64.0% to 80.0%, while the sensitivity remained high at 100.0%. The positive predictive value (PPV) was 19.7% [8].

Recent guidelines recommend additional imaging with another modality if an adrenal mass is indeterminate on noncontrast CT (>10 HU) [4]. One such modality is contrast-enhanced CT. There is an assumption that ACAs have the property of early enhancement after contrast administration and rapid loss of contrast, unlike nonadenomas. This makes the calculation of wash-out rates useful in diagnosing lipid-poor adenomas. A relative washout (RPW) of >40% and an absolute washout (APW) of >60% are commonly used to suggest that an adrenal lesion is benign [4]. However, data supporting this assumption are scarce, and they have been shown to lead to misdiagnosis [13]. According to a recent study by Schloetelburg et al., when using the above-mentioned thresholds, only 69% (by RPW) and 64% (by APW) of ACAs were classified correctly. Additionally, four to eleven out of forty-nine potentially malignant adrenal tumors were misdiagnosed. The study suggests a cut-off of 58% for RPW to detect all malignant tumors, but this resulted in a low specificity of only 15% [15]. Benign oncocytic adrenal neoplasms (OANs) do not differ from ACCs or malignant OANs in terms of nonenhanced CT attenuation values. This is due to the low lipid content of oncocytic tumors in general. However, the percentage of APW in benign OANs is noticeably higher than in ACCs and malignant OANs [16].

Studies have shown that the size of an adrenal mass is correlated with the risk of malignancy. In the EURINE-ACT study, which prospectively recruited patients with newly diagnosed adrenal masses, 98% of ACCs had a tumor diameter of ≥4 cm, and 2% had a diameter of ≥2 to <4 cm. None had a diameter <2 cm [8]. These data are consistent with other studies, where ACCs with a diameter less than 5 cm were observed in 3% of cases and less than 6 cm in 4.2% of cases [10,11]. The median diameter at the time of diagnosis varied from 7.5 cm [7] to 11.4 cm [17].

An increase in size during follow-up is typically considered a feature of malignancy. However, a study by Barzon et al. found that mass enlargement of greater than 1 cm and/or the appearance of another mass in the contralateral adrenal gland over a mean period of 3 years occurred in 9% of cases of incidentalomas. The risk of malignancy over time for masses defined as benign at diagnosis is estimated to be about 1/1000 [2].

## 4. Magnetic Resonance Imaging (MRI)

MRI is a complementary diagnostic tool for evaluating adrenal tumors and is the method of choice in cases where there are contraindications to the use of iodine-containing contrast agents. The basis for radiological evaluation in differentiating between ACC and ACA is the assessment of the tumor signal using chemical shift imaging, specifically T1-weighted images in phase and opposed-phase. ACA with high lipid content loses signal intensity in the opposed-phase image, while in the case of ACC, the signal remains unchanged or is only partially reduced. The signal loss ratio is one of the quantitative tools used to assess signal intensity, but it does not provide a definitive answer as to the nature of the tumor. MRI is particularly useful in the evaluation of lipid-poor adenomas [4,12,18].

According to the analysis by Dinnes J., MRI has a slightly lower sensitivity and specificity compared with CT in detecting incidentalomas. However, in the case of focal lesions in patients with current or prior nonadrenal malignancy, the sensitivity of MRI was found to be 89–90%, with a specificity of 60–93% [13]. Therefore, this study can be specifically useful for patients suspected of having metastatic lesions in the adrenal glands.

## 5. Positron Emission Tomography (PET)

PET with 18F-fluorodeoxyglucose is the most commonly performed PET test in the diagnosis and monitoring of malignancies of various origins. The degree of tracer accumulation depends primarily on the metabolic activity of the tumor cells, which is why rapidly growing malignant lesions and benign endocrine tumors with intense secretory activity show enhanced uptake. PET has been used to diagnose adrenal lesions in patients with confirmed metastatic lesions in other locations [13]. According to reports, the use of PET to distinguish between adrenal lesions such as pheochromocytoma, metastasis, and adenoma does not always yield clear results. In fact, it is estimated that only about 5% of cases would benefit from this test and have a change in clinical management [19,20]. PET should only be used when there is a high likelihood of an extra-adrenal malignant tumor [21].

## 6. Texture

Tumors exhibit heterogeneity at genetic and histopathological levels, including variations in cellular density, angiogenesis, extracellular matrix composition, and the presence of necrotic areas. High intratumoral heterogeneity has been associated with a poorer prognosis, potentially due to more aggressive biology. Tumor heterogeneity poses challenges for capturing and quantifying with traditional imaging tools, as a common limitation across imaging modalities is that visual interpretation relies on the human eye, which may not readily appreciate certain features within the images [22,23]. This limitation has led to the development of texture analysis (TA), which is an emerging field of radiomics. TA utilizes mathematical algorithms to extract information about the spatial distribution of signal intensities and pixel interrelationships within medical images and analyzes the data using artificial intelligence methods. TA aims to quantify textural information that reflects the underlying heterogeneity of the tumor, providing clinicians with additional data to aid in diagnosis and treatment planning [24].

The research findings suggest that there is a positive correlation between the heterogeneity of a lesion and the risk of malignancy. Elmohr et al. found that ACCs exhibit increased attenuation (measured by gray level) and heterogeneity compared with ACAs [25]. Crimì et al. performed a systematic review on the application of TA in adrenal masses; the pooled median area under the ROC curve across all studies was 0.85, indicating high diagnostic accuracy. Notably, the differentiation between ACA and ACC achieved a diagnostic accuracy of up to 93%. These findings are very promising; however, the method needs further validation [26].

## 7. Hormonal Evaluation

ACA and ACC can either be hormonally inactive or produce an excess of hormones, leading to specific clinical syndromes. Roughly 45.7% to 59.3% of the ACC cases are hormonally active [11,27]. However, in the study where ACC was found incidentally, only 10% of cases were steroid-producing [7].

It is recommended that patients with suspected ACC undergo a thorough clinical assessment and endocrine workup. This includes measuring free cortisol in 24-h urine, basal levels of adrenocorticotropic hormone (ACTH), dehydroepiandrosterone sulfate (DHEA-S), 17-OH progesterone (17HP), androstenedione, and testosterone (only in women), as well as 17-beta-estradiol (only in men and postmenopausal women), 11-deoxycortisol (if available), potassium, and aldosterone/renin ratio (for patients with hypertension or unexplained hypokalaemia). Lastly, a 1 mg dexamethasone suppression test should be performed [5,28].

Since conventional imaging studies cannot distinguish ACC from pheochromocytoma, it is recommended to determine fractionated metanephrines in 24 h urine or free plasma-metanephrines in any case of adrenal tumor when there is no obvious autonomic steroid excess. An unrecognized pheochromocytoma can lead to dangerous hypertensive crises (especially during invasive procedures). However, it should be noted that slightly elevated levels of metanephrines (<2-fold), especially when not consistent with large tumor size, may be nonspecific and can be observed in ACC [5].

Steroid hormone profiling can aid in assessing the risk of malignancy in ACCs. According to the data presented by Arlt et al., routine biochemistry tests indicated that 32% of ACA patients and 73% of ACC patients had evidence of hormone excess [29]. The study group included forty-four patients with ACC, eleven of whom had isolated glucocorticoid access, seven had adrenal androgen excess, and twelve had combined glucocorticoid and adrenal androgen excess, with two of them exhibiting additional aldosterone excess. Additionally, 56% of ACC patients had elevated 17-HP levels [29].

Studies indicate that patients with ACC have higher levels of DHEA-S than those with ACA. Elevated levels of DHEA-S were found in 2–3% of ACAs compared with 17–42% of ACCs [7,29,30]. However, the sensitivity and specificity of high DHEA-S as a marker for ACC were found to be 17% and 93%, respectively [7].

## 8. Urine Steroid Metabolite Profiling

Urine steroid metabolomics is a diagnostic method that involves analyzing the levels of different steroid hormones and their precursors in urine through liquid chromatography–tandem mass spectrometry (LC–MS/MS).

According to a study by Arlt et al., urine steroid metabolite profiling has the capacity to differentiate between ACC and ACA with a sensitivity and specificity of 90% [29]. The results of the study suggest that combined androgen and glucocorticoid excess is a characteristic feature of ACC, which can be detected with higher sensitivity through urinary steroid profiling than routine biochemistry (69% of the cases by urine metabolite profiling vs. 27% of the cases by routine biochemistry). Additionally, the 11-deoxycortisol metabolite (tetrahydro-11-deoxycortisol) was found to be the most effective at distinguishing between ACC and ACA [29]. Increased levels of androgen precursors in the urine were found in 71% of ACC patients and 2% of ACA patients, whereas increased levels of DHEAS in the serum were only found in 42% of ACC patients and 2% of ACA patients. Interestingly, 27% of ACCs that were classified as hormonally inactive based on routine biochemistry exhibited significant steroid precursor excess [29].

In a recent multicenter study, the EURINE-ACT trial, urine steroid metabolomics profiles indicating a high risk of ACC were observed in 84.7% of the 98 participants with ACC and in 8.2% of the 1919 participants with nonACC masses. This method significantly improved the positive predictive value compared with imaging tests [8]. A low-risk score was also found to have the same ability as imaging tests to rule out ACC. The triple testing strategy involved evaluating three factors: tumor diameter larger than 4 cm, positive imaging characteristics (attenuation greater than 20 HU), and a urine steroid metabolomics result indicating a high risk of ACC. The triple testing strategy identified a group of 106 participants, including 81 of the 98 participants with ACC, yielding a positive predictive value for ACC of 76.4%. The diagnostic accuracy of urine steroid metabolomics was found to be higher than that of maximum tumor diameter and imaging characteristics; the best performance was observed when these three methods were combined [8].

## 9. Liquid Biopsies (LB)

The use of liquid biopsies (LB) to diagnose ACC involves detecting neo cells and “cell-free” nucleic acids (DNA, RNA, and microRNA) that are derived from the tumor and released into the blood and other body fluids using modern molecular techniques such as PCR and next-generation sequencing [31].

Pinzani et al. assessed the presence of circulating tumor cells (CTCs) in patients with ACC and ACA. Their study, although conducted on a small cohort of patients (14 patients with ACC and 10 with ACA), provides a lot of valuable information. CTCs were detected in all patients with ACC and none with ACA; a significant decrease in CTCs was observed after surgery; and a correlation between tumor size and CTC concentration was demonstrated in the research [32].

Circulating tumor DNA (ctDNA) is another marker that can be relevant in non-invasive diagnostics of different types of cancer [31]. A higher amount of ctDNA was found in advanced stages of different cancers in relation to locoregional stages [33]. However, the number of studies evaluating the usefulness of ctDNA in patients with ACC is small, and the study groups are insufficient to draw clear conclusions [31]. Creemers et al. detected tumor-specific ctDNA mutations in only one of three patients with mutations in primary ACC (the study group consisted of six patients) [34]. In another study of sixteen patients with ACC, ctDNA mutations indicating a malignant DNA origin were identified in only two patients [35].

MicroRNAs (miRNAs) are endogenous, small, non-coding RNA molecules involved in the regulation of gene expression [36]. MiRNAs are stable molecules whose expression can be assessed not only in tissues but also in body fluids, including plasma [37]. Xi Chen and al. identified specific expression patterns of serum miRNAs for lung cancer, colorectal cancer, and diabetes, providing evidence that serum miRNAs contain fingerprints for various diseases [38]. Several circulating blood-borne miRNAs have been evaluated as potential differential markers between ACA and ACC, with miR-483-5p, significantly overexpressed in the ACC vs. ACA group, appearing to be the most promising [39]. However, one must remember that the data are not certain. The sensitivity and specificity values of miRNA markers were variable, and in one of the studies, no significant differences were found in the expression of miR-483-5p between adrenal myelolipoma (AML) and the ACC group [39,40].

While LB shows promise, it is still in the early stages and requires further research, especially in the context of ACC diagnosis [31].

## 10. Pathological Workup

Histopathology obtained from resected adrenals is considered the gold standard for diagnosing ACC [5]. However, needle adrenal biopsies are generally not recommended due to the high risk of nondiagnostic results (up to 28%) and potential complications (with a pooled rate of 2.5%) [41]. Nonetheless, adrenal tumor biopsy may be considered in certain situations, such as when a patient is not a candidate for surgery (inoperable ACC) or when there is a high suspicion of extra-adrenal metastasis [5]. The presence of a catecholamine-secreting tumor should be considered before proceeding with a biopsy. Biochemical evaluation (urinary or serum metanephrine concentrations) is necessary even if the clinical picture indicates a high oncologic risk [41]. Inadvertent biopsy can lead to serious consequences of excess catecholamines—hypertensive crisis, myocardial infarction, and uncontrolled bleeding with fatal outcomes [6]. Adrenal biopsies can be performed by percutaneous or endoscopic routes. Abdominal ultrasonography (US) and CT scans are the most commonly used imaging modalities for this procedure. US-guided biopsy enables real-time workup, while CT provides new and more accurate information about adjacent organs. An important limitation of US-guided biopsy is the poor visibility of adrenal tumors, especially on the left side, while CT biopsy exposes the patient to radiation and is much more expensive. The choice of the procedure is multifactorial, depending on the size and location of the lesion, the availability of the method, and the doctor’s and patient’s approval [42,43]. Endoscopic ultrasound-guided fine needle aspiration (EUS-FNA) is an advanced procedure that allows a biopsy of the adrenal glands. It has been proven in the literature that EUS-FNA can be mainly beneficial in the invasive diagnosis of lesions located on the left adrenal gland (due to technical difficulties in reaching the right side). The procedure can be particularly applicable in significantly advanced adrenal tumors as it allows for additional cytological samples, e.g., from the gastrointestinal tract and mediastinal lymph nodes. The limitations of EUS-FNA are primarily cost, the need for appropriate equipment, and qualified medical personnel [44,45]. Relative contraindications to image-guided adrenal biopsy include uncorrectable coagulopathy, the inability to reach the tumor via a safe path, or an unsafe target [46].

The diagnostic performance of adrenal biopsy in detecting adrenal metastatic disease was found to be 87–88.8% sensitive and 93.6–96% specific [41,47]. In comparison, the sensitivity of adrenal biopsy in diagnosing ACC is around 70% [48]. This may be due to the difficulty in distinguishing between ACA and ACC, even when the entire tumor specimen is available for examination. Additionally, biopsy procedures may yield insufficient tissue material to apply all the criteria for the Weiss score system [41].

The Weiss score is the most widely employed scoring system to distinguish between ACC and ACA. It is based on nine histological criteria, with a score of three or higher indicating ACC [49]. Studies have shown that the Weiss score has a sensitivity of 94–100% and a specificity of 96–97% in predicting malignancy [50,51]. Table 2 shows the histological criteria used to assess the malignant potential of adrenocortical tumors [3,52].

Necrosis was found to be the most sensitive feature of the Weiss score in a study by Volante et al., which was detected in 84% of ACC cases, followed by a mitotic count exceeding five in fifty HPF (71%) and venous invasion (64%) [17]. Some authors have highlighted that some of the Weiss criteria are difficult to interpret or subject to interobserver variability, leading to efforts to simplify the score. A modified Weiss system includes only five criteria (shown in Table 3) and has been found to be easier to use in practice and has a significant correlation with the Weiss system [50].

ACA typically lacks all Weiss parameters, while most cases of ACC have a Weiss score of six or higher, indicating a high likelihood of malignancy [53]. In some instances, a diagnosis of malignancy may not be definitive even if two or three Weiss parameters are present and the final score is not high enough. Such cases are categorized as borderline tumors or tumors of uncertain malignant potential (UMP) since they imply the possibility of malignancy [53]. The Weiss system includes a “gray zone” where tumors with a score of two or three are potentially malignant. However, some studies have reported that tumors with a Weiss score of three may not necessarily recur, while others have reported that tumors with a score of two may recur with metastases [54,55]. A study conducted by Duregon et al. reviewed 300 consultation cases of histopathological specimens. During the consultation, nine cases of adrenocortical tumors were reinterpreted, with four carcinomas being reclassified as adenomas and five adenomas being reclassified as carcinomas [56]. Interestingly, out of the five adenomas that were reclassified as carcinomas, four were oncocytic tumors. Eight cases (2.6%) were submitted for revision with a diagnosis of UMP. Out of these cases, seven were oncocytic, and all of them were classified as UMP according to the Lin-Weiss-Bisceglia system, which is a modification of the Weiss score [56]. The accuracy of these scores has been a matter of debate, with several pitfalls and controversies surrounding their correct application. This is particularly true for the oncocytic variant, where the definition of malignancy in oncocytic adrenocortical tumors (OACTs) could be problematic when using the classic Weiss score and may lead to an overestimation of their malignancy potential. This is due to the fact that some of the classical Weiss parameters, such as eosinophilic cytoplasm and nuclear atypia, are present in many OACTs regardless of their biological behavior [57,58]. It is becoming increasingly clear that variants of ACC, especially OACT, differ from conventional ACC not only in terms of their morphology but also in terms of their molecular features, prognosis, and possibly their response to treatment [56,58,59,60].

Multiple studies have demonstrated the accuracy of the Ki67 index for diagnosing ACC, and current guidelines recommend its use in evaluating resection specimens of adrenal cortical tumors [5]. A Ki67 index of 4% has been shown to have a sensitivity of 95.7% and a specificity of 91.7% for diagnosing ACC [50]. The cut-off for assessing malignancy based on the Ki67 index is typically set at 5%. A proliferation index of >5% is generally only observed in the ACC. However, it is possible for ACC to have a low proliferation index, similar to what is observed in ACA [61]. Additionally, it is crucial to note that the Ki67 proliferation index can vary within different regions of a tumor, with some areas exhibiting higher levels of proliferation than others (known as “hot spots”). Therefore, assessing only a biopsy sample may not accurately represent the entire tumor and could potentially be misleading [5].

The pathology report should include the Weiss score, Ki67 index, resection status, and pathological tumor stage (which indicates whether the tumor has invaded the capsule and/or surrounding tissue and organs). Additionally, nodal status should be included. These recommendations are still present in the current guidelines [5].

## 11. Role of Selected Adipokines in Differentiation between ACC and ACA

While obesity is a well-known risk factor for various types of cancer, there is growing evidence to suggest that adipose tissue and its secreted hormones and cytokines, known as adipokines, may play a role in carcinogenesis even in non-obese individuals [62,63,64].

Adiponectin is a well-known adipokine that activates several intracellular signaling pathways, including 5′-AMP-activated protein kinase (AMPK), mammalian target of rapamycin (mToR), nuclear factor κB (NF-kB), c-Jun N-terminal kinase (JNK), and signal transducer and activator of transcription proteins (STATs) [65,66]. Adiponectin is believed to be a mediator of obesity-related cancers and exerts direct anticancer effects via its receptors [67]. The protective effect of adiponectin on cancer is based on its anti-proliferative properties [68]. Chou et al. investigated the expression of adiponectin receptors in various human neoplasms, including non-obesity-associated human neoplasms, and the authors were the first to demonstrate their presence in ACC [63]. The authors of the current review evaluated differences in the expression of adiponectin receptors in benign and malignant adrenal tumors using immunohistochemistry. In an analysis of 128 resected adrenal tumors, both AdipoR1 and AdipoR2 expression levels were significantly higher in ACC compared with benign adrenal tumors [69]. The higher expression of adiponectin receptors in malignant adrenal tumors may be due to a down-regulation mechanism that has been observed and studied in epithelial breast cancer and Barrett’s adenocarcinoma [62]. It might be tentatively speculated that the low serum levels of adiponectin, which can be related to a proneoplastic influence, result in an increased expression of Adipo R1 and R2 receptors in the adrenal tumor tissue, which is evident in their higher expression in ACC [62].

Leptin is another adipokine that may play an important role in carcinogenesis. This adipokine promotes proliferation via a variety of growth factors such as vascular endothelial growth factor (VEGF), fibroblast growth factor 21 (FGF21), and IGF-1 [70,71]. Leptin has been shown to increase the growth of some extra-adrenal neoplasms such as breast, esophageal, stomach, pancreatic, prostate, ovarian, and lung cancer cells [72]. In the above-mentioned study conducted by the authors of this review, it was demonstrated that leptin receptor expression was absent or minimal in half of the examined benign tumors while being much higher in ACCs [69].

However, the exact role of adiponectin and leptin receptors in assessing the risk of malignancy or differentiation between ACC and ACA is still unknown, and further prospective research is required.

Visfatin, also known as nicotinamide phosphoribosyltransferase (NAMPT) or pre-B-cell-enhancing factor, is a newly discovered adipocytokine expressed mostly in visceral adipose tissue. Based on a meta-analysis by Mohammadi et al., higher levels of visfatin are associated with a higher risk of developing cancer [73]. Visfatin stimulates the production of many proinflammatory cytokines and enhances the production of IL-1, IL-6, IL-8, and TNF α [74]. In the apoptotic state, visfatin-induced antioxidative activity results in increased viability of the cancer cells [74]. Another mechanism that enhances cancer cells survival is participation in the NAD generation pathway [74]. The authors of the recent study found that patients with ACC had significantly higher serum levels of visfatin compared with patients with ACAs, and a visfatin serum concentration of 8.05 ng/mL or higher could potentially be used to discriminate between the two with a sensitivity of 50.0% and a specificity of 92.3% [75]. This is the first research study studying the role of visfatin in differentiation between ACC and ACA. While the results of the study are promising, further research and validation in larger groups of patients are required.

## 12. Conclusions

Although there are typically distinguishable features between benign and malignant adrenal masses, a significant number of tumors fall into a gray zone between ACA and ACC, making diagnosis challenging. These cases require additional testing and often follow-up examinations to establish an accurate diagnosis and predict the course of the disease. By using a combination of noncontrast CT imaging, tumor size, and metabolomics evaluation, it is possible to determine the nature of the tumor with high accuracy before deciding on a surgical treatment. This approach can help narrow down the group of patients who have adrenal tumors and require surgical treatment due to the suspected malignant nature of the lesion. Unnecessary adrenalectomy can be associated with complications, psychological burden for patients, and additional costs for healthcare systems around the world.

## Figures and Tables

**Table 1:** Etiology and incidence of adrenal incidentaloma.

Etiology	Barzon et al. [2]	Bednarczuk et al. [3]
Adrenal cortical tumors ■ adenoma ■ nodular hyperplasia ■ carcinoma	36–94 7–17 1.2–11	~80% ~5%
Adrenal medullary tumors ■ pheochromocytoma ■ ganglioneuroma	1.5–23 0–6	~5%
Other adrenal tumors ■ myelolipoma ■ lipoma ■ other	7–15 0–11 <1	
Cysts and pseudocysts	4–22	
Hematoma and hemorrhage	0–4	
Metastases	0–21	~2%
Pseudoadrenal masses	0–10	

**Table 2:** Histological criteria for assessment of malignant potential in adrenocortical tumors [3,52] (modified).

High nuclear grade (III or IV)
Mitotic rate (at least six per fifty high-power fields)
Atypical mitoses
Clear cells ≤25 of the tumor
Diffuse architecture more than one-third in tumor
Necrosis
Venous invasion
Sinusoidal invasion
Capsular invasion

**Table 3:** Modified Weiss system [3,52] (modified).

Mitotic rate (at least six per fifty high-power fields)
Atypical mitoses
Clear cells ≤25 of the tumor
Necrosis
Sinusoidal invasion
Capsular invasion

## Data Availability

Not applicable.

## Abbreviations

ACA	adrenocortical adenoma
ACC	adrenocortical cancer
ACTH	adrenocorticotropic hormone
AML	adrenal myelolipoma
AMPK	5′-AMP-activated protein kinase
APW	absolute washout
CT	computed tomography
CTCs	circulating tumor cells
ctDNA	circulating tumor DNA
DHEA-S	dehydroepiandrosterone sulfate
EUS-FNA	endoscopic ultrasound-guided fine needle aspiration
FGF21	fibroblast growth factor 21
HU	hounsfield units
IGF-1	insulin-like growth factor I
IL	interleukin
JNK	Jun N-terminal kinase
LB	liquid biopsies
LC–MS/MS	liquid chromatography–tandem mass spectrometry
miRNAs	microRNAs
MRI	magnetic resonance imaging
mTOR	mammalian target of rapamycin (NF-kB)
NAD	nicotinamide adenine dinucleotide
NF-kB	nuclear factor κB
OANs	oncocytic adrenal neoplasms
PET	positron emission tomography
PPV	positive predictive value
RPW	relative washout
STATs	signal transducer and activator of transcription proteins
TA	texture analysis
TNF	tumor necrosis factor
UMP	tumors of uncertain malignant potential
US	ultrasonography
VEGF	vascular endothelial growth factor
17HP	17-hydroxyprogesterone
